# Unveiling the Assembly of Neutral Marine Polysaccharides into Electrostatic-Driven Layer-by-Layer Bioassemblies by Chemical Functionalization

**DOI:** 10.3390/md21020092

**Published:** 2023-01-27

**Authors:** Luís P. G. Monteiro, João Borges, João M. M. Rodrigues, João F. Mano

**Affiliations:** Department of Chemistry, CICECO—Aveiro Institute of Materials, University of Aveiro, 3810-193 Aveiro, Portugal

**Keywords:** marine-origin polysaccharides, laminarin, pullulan, peptides, electrostatic-driven layer-by-layer assembly, biocompatible multilayered thin films

## Abstract

Marine-origin polysaccharides, in particular cationic and anionic ones, have been widely explored as building blocks in fully natural or hybrid electrostatic-driven Layer-by-Layer (LbL) assemblies for bioapplications. However, the low chemical versatility imparted by neutral polysaccharides has been limiting their assembly into LbL biodevices, despite their wide availability in sources such as the marine environment, easy functionality, and very appealing features for addressing multiple biomedical and biotechnological applications. In this work, we report the chemical functionalization of laminarin (LAM) and pullulan (PUL) marine polysaccharides with peptides bearing either six lysine (K_6_) or aspartic acid (D_6_) amino acids via Cu(I)-catalyzed azide-alkyne cycloaddition to synthesize positively and negatively charged polysaccharide-peptide conjugates. The successful conjugation of the peptides into the polysaccharide’s backbone was confirmed by proton nuclear magnetic resonance and attenuated total reflectance Fourier-transform infrared spectroscopy, and the positive and negative charges of the LAM-K_6_/PUL-K_6_ and LAM-D_6_/PUL-D_6_ conjugates, respectively, were assessed by zeta-potential measurements. The electrostatic-driven LbL build-up of either the LAM-D_6_/LAM-K_6_ or PUL-D_6_/PUL-K_6_ multilayered thin film was monitored in situ by quartz crystal microbalance with dissipation monitoring, revealing the successful multilayered film growth and the enhanced stability of the PUL-based film. The construction of the PUL-peptide multilayered thin film was also assessed by scanning electron microscopy and its biocompatibility was demonstrated in vitro towards L929 mouse fibroblasts. The herein proposed approach could enable the inclusion of virtually any kind of small molecules in the multilayered assemblies, including bioactive moieties, and be translated into more convoluted structures of any size and geometry, thus extending the usefulness of neutral polysaccharides and opening new avenues in the biomedical field, including in controlled drug/therapeutics delivery, tissue engineering, and regenerative medicine strategies.

## 1. Introduction

Over the last three decades, the Layer-by-Layer (LbL) assembly technology has proven to be an easy, cost-effective, and highly versatile technology to coat surfaces and develop a wide array of (multi)functional multilayered assemblies across multiple length scales with tunable compositions, structures, properties, and functions at the nanoscale [1,2,3]. The simplicity and versatility imparted by the LbL technology are unveiled by the alternate and sequential adsorption of a wide array of building blocks exhibiting complementary intermolecular interactions on virtually any type of surface, from flat planar to more convoluted geometries, by resorting to a variety of deposition methodologies [1,2,4,5]. Dipping [6,7,8], spin-coating [9,10], and spraying [11,12,13,14] have been the most widely used methodologies to assemble multilayered films on surfaces, each with its advantages and disadvantages [2,3]. Among the multitude of intermolecular interactions, the electrostatic interaction between oppositely charged polyelectrolytes is by far the most explored mechanism behind the fabrication of multilayered assemblies, whose properties and functions can be finely tuned by playing with numerous parameters. These include the intrinsic properties of the adsorbed molecules (e.g., composition, structure, and charge density), the physicochemical properties of the liquid medium (e.g., pH, ionic strength, buffer composition, and solvent quality), and external parameters (e.g., temperature, exposure to light, electrical field, adsorption time, and the number of adsorbed layers) [15,16]. However, since the LbL assembly process can occur under mild conditions in entirely aqueous solutions, the electrostatic interaction has been extended well beyond the assembly of oppositely charged polyelectrolytes. In fact, an unlimited number of charged materials, including biomolecules such as marine polysaccharides [17,18,19,20], proteins [21], enzymes [22,23], nucleic acids [24], or growth factors [25], which have limited solubility in nonaqueous solutions and may lose their biological activity, have been processed into electrostatic-driven functional multilayered assemblies for addressing a wide variety of biomedical and biotechnological applications. In particular, marine polysaccharides, which are widely discarded as waste in the processing of marine organisms, are particularly attractive ingredients for enabling the assembly of sustainable biomaterials in an LbL fashion owing to their very appealing features [26]. Those include their biocompatibility, biodegradability, non-toxicity, non-immunogenic, antitumoral and anticoagulant properties, chemical versatility, and wide availability [27,28]. For instance, chitosan (CHT), a positively charged natural biopolymer obtained by the deacetylation of chitin extracted from crab and shrimp shells, has been extensively combined with structurally similar yet oppositely charged polysaccharides [17,29,30], proteins [31,32], and even other biomolecules [33], in order to shape a plethora of LbL structures that hold great potential to be applied in the biomedical arena. However, to the best of our knowledge, the assembly of neutral marine polysaccharides into LbL biostructures has not been exploited to date, despite their wide abundance and variety, easy functionality, and invaluable potential in the biomedical and biotechnological fields. For instance, laminarin (LAM) — a low-molecular-weight, branched, and neutral marine polysaccharide found in brown algae, which is composed of glucose units linked by *β*-1,3 glycosidic bonds, as well as by some *β*-1,6 side-chain branches — has been recently functionalized with a wide variety of chemical functional groups for being processed into either photocrosslinkable hydrogels or microparticles with high stability for being used in 3D cell culture, tissue engineering, and regenerative medicine [34,35,36,37,38]. Pullulan (PUL) is a high-molecular-weight, linear, and neutral polysaccharide composed of maltotriose units linked by *α*-1,4-bonds produced by the yeast-like fungus (*Aureobasidium pullulans*), which can be found in several marine environments, from marshes to marine sediments and estuarine waters [39]. PUL has been chemically modified and processed into hydrogels, scaffolds, sponges, and nanofiber meshes to be used in a wide variety of biomedical applications, including as platforms for cell culture, wound dressings, or as injectable systems [40,41,42,43,44,45]. However, to date, there are no studies that report on either the covalent-driven LbL assembly of such marine-origin polysaccharides or their covalent functionalization with charged pendant functional groups to enable their assembly into electrostatically driven multilayered assemblies. The latter are particularly attractive for bioapplications, owing to their susceptibility to being made responsive to a variety of stimuli, thus enabling the assembly of smart systems.

Herein, we explored the chemical functionalization of LAM and PUL with peptide sequences denoting six oppositely charged amino acids [either lysine (K_6_) or aspartic acid (D_6_)] as a straightforward way to enable the construction of either wholly LAM or PUL-derived multilayered thin films by resorting to LbL assembly technology. To the best of our knowledge, this is the first time that such uncharged polysaccharides have been functionalized with peptides bearing charged functional groups to enable their processing into electrostatic-driven multilayered assemblies. Both polysaccharides were first chemically functionalized with azide moieties for further coupling them to peptides exhibiting alkyne groups via Cu(I)-catalyzed azide-alkyne cycloaddition (CuAAC), as evaluated and characterized by proton nuclear magnetic resonance (^1^H NMR) and attenuated total reflectance Fourier-transform infrared (ATR-FTIR) spectroscopy. The charge densities of the positive and negatively charged polysaccharide-peptide conjugates were measured by zeta-potential, and their possible interaction and multilayer film growth were studied in real-time by quartz crystal microbalance with dissipation monitoring (QCM-D). The morphological properties of the most stable multilayered thin film were investigated by scanning electron microscopy (SEM) and their in vitro biological performance was studied to prove their biocompatibility and suitability for being used in bioapplications. We foresee that this proof-of-concept work will launch the seeds for further studies in which virtually any neutral biopolymers could be used. In this regard, the herein developed multilayered assemblies and other combinations of functionalized neutral biopolymers could be employed in coating substrates of any size, shape, and surface chemistry, thus enabling the assembly of a diverse set of multilayered devices for addressing a wide array of biomedical applications.

## 2. Results and Discussion

### 2.1. Synthesis and Physicochemical Characterization

**Figure 1:**
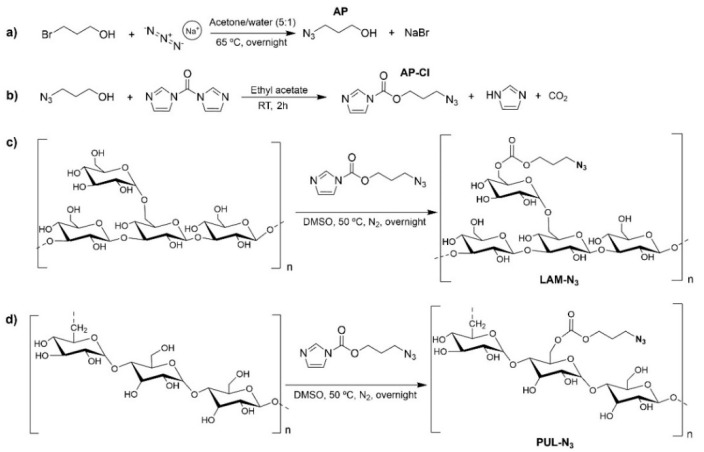
Reaction scheme of the synthetic route to obtain the: (**a**) 3-azidopropanol (AP) and (**b**) 3-azidopropyl carbonylimidazole (AP-CI), and chemical modification of (**c**) LAM and (**d**) PUL with azide moieties to obtain the LAM-N_3_ and PUL-N_3_ compounds.

The well-established CuAAC reaction was employed to couple the charged peptides, containing an alkyne functional group, to the neutral polysaccharides bearing azide moieties. To chemically modify the polysaccharide’s backbone with azide moieties, we first synthesized the 3-azidopropyl carbonylimidazole (AP-CI) compound, as described elsewhere [38]. Firstly, AP (Figure 1a) was successfully synthesized by nucleophilic substitution from the azide anion and the alkyl halide in 69.4% yield. The chemical structure of the synthesized AP was confirmed by ^1^H NMR spectroscopy, where it is possible to observe the presence of the aliphatic protons at 1.84, 3.46, and 3.76 ppm (Figure S1a). Next, AP-CI was obtained with a 65.3% yield by reacting AP with 1,1’-carbonyldiimidazole (CDI) (Figure 1b) [38]. The final structure was confirmed by ^1^H NMR spectroscopy, as showcased by the presence of the aliphatic protons at 1.99, 3.55, and 4.43 ppm, and the aromatic protons of the diazole ring that appear in the form of two multiple signals at 7.08, 7.63, and 8.30 ppm (Figure S1b).

**Figure 2:**
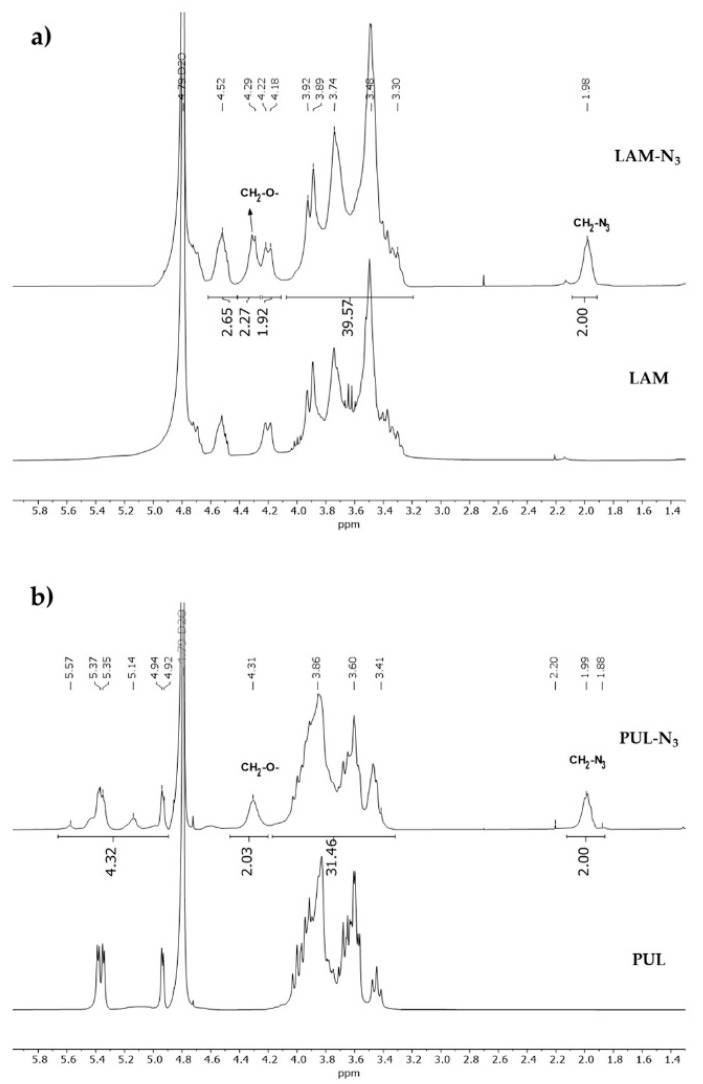
^1^H NMR spectra of: (**a**) LAM and LAM-N_3_ and (**b**) PUL and PUL-N_3_, in D_2_O.

LAM-N_3_ and PUL-N_3_ were then synthesized with a degree of substitution (DS) of 17 and 21%, respectively. Both modified neutral polysaccharides were obtained upon reaction with AP-CI in DMSO, which resulted in the formation of a carbonate ester between the polysaccharide backbone and the azidopropyl moiety (Figure 1c,d). The successful grafting of the azide groups in the polysaccharide’s backbone was confirmed by ^1^H NMR spectroscopy, where it is possible to observe differences between the native and the polysaccharide derivatives. In both polysaccharide derivatives, LAM-N_3_ (Figure 2a) and PUL-N_3_ (Figure 2b), the ^1^H NMR spectra clearly show the presence of the aliphatic protons, as denoted by the appearance of the resonance signals of CH_2_-N_3_ protons at 1.98 ppm and of CH_2_-O protons around 4.30 ppm, thus confirming the successful functionalization of the polysaccharides with azide moieties.

**Figure 3:**
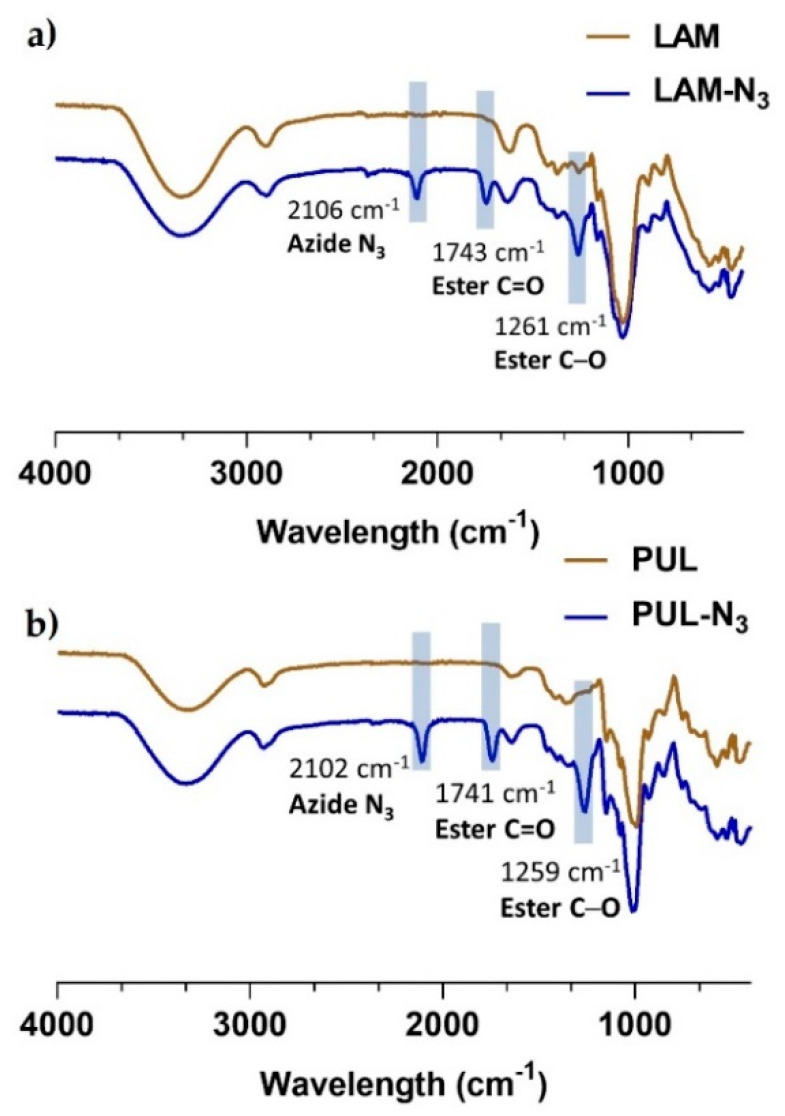
ATR-FTIR spectra of: (**a**) LAM and LAM-N_3_ and (**b**) PUL and PUL-N_3_. Highlighted are the vibrations bands of the azide groups and esters.

Attenuated total reflectance Fourier-transform infrared spectroscopy (FTIR-ATR) was also performed with dry powders to validate the successful modification of the polysaccharides. The ATR-FTIR spectra of LAM-N_3_ and PUL-N_3_ (Figure 3) showcase well-defined vibration bands at 2106 cm^−1^ (LAM-N_3_) and 2102 cm^−1^ (PUL-N_3_), which are not seen in the spectra of the native polysaccharides, thus proving the successful functionalization of both biopolymers with azide groups. Moreover, the vibration bands of the carbonate ester bonds formed between the polysaccharide backbone and the azide moieties are denoted at *ca.* 1261 cm^−1^ and 1741 cm^−1^. These results are in line with the ^1^H NMR data, confirming the successful chemical modification of the polysaccharides with azide moieties.

**Figure 4:**
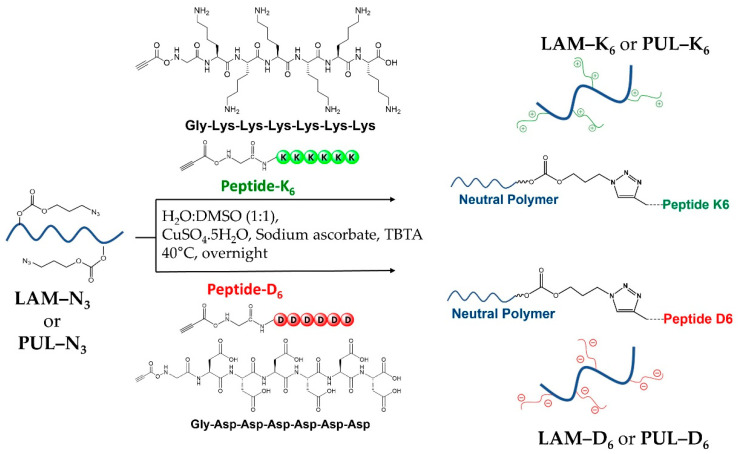
Schematic representation of the chemical route used to couple the charged peptides to the marine polysaccharides via CuAAC.

**Figure 5:**
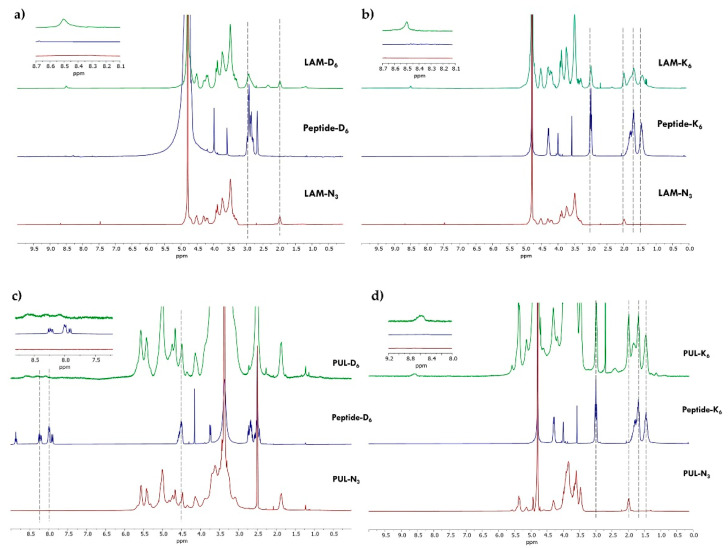
^1^H NMR spectra of: (**a**) LAM-N_3_, Peptide-D_6_, and LAM-D_6_ in D_2_O; (**b**) LAM-N_3_, Peptide-K_6_, and LAM-K_6_ in D_2_O; (**c**) PUL-N_3_, Peptide-D_6_, and PUL-D_6_ in DMSO-*d*_6_ (**d**) PUL-N_3_, Peptide-K_6_, and PUL-K_6_ in D_2_O. The dashed lines highlight the peptide proton peaks that are also present in the polysaccharide-peptide conjugates.

To obtain the novel polysaccharide-peptide conjugates, CuAAC coupling chemistry was applied to couple the alkyne-modified peptides to the polysaccharides-N_3_, as schematically illustrated in Figure 4, ensuring high selectivity [46,47]. The successful conjugation through CuAAC reaction was confirmed by ^1^H NMR spectroscopy, where it is evident the presence of the proton peak corresponding to the triazole ring at 8.5 ppm (Figure 5). It is also noteworthy the presence of characteristic peptide proton peaks in the spectra of the polysaccharide-peptide conjugates. The ^1^H NMR spectra of both peptides were also acquired in D_2_O and analyzed separately (Figure S2).

**Figure 6:**
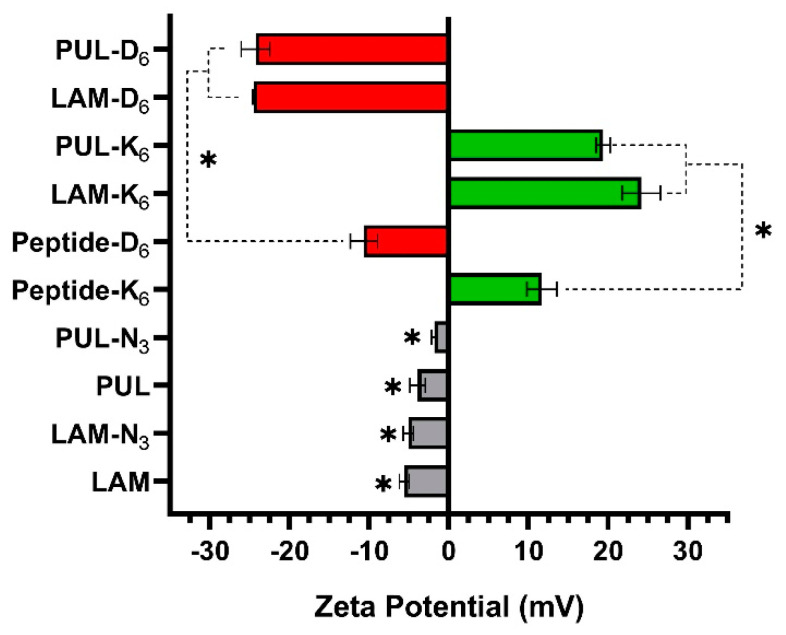
The ζ-potential measurements (*n* = 3) of the aqueous solution of the polysaccharide-peptide conjugates at 0.5 mg.mL^−1^ in 0.1 M PBS at pH 7.4. For comparison, the native polysaccharides (LAM and PUL), the precursors polysaccharides-N_3_ (LAM-N_3_ and PUL-N_3_), and the peptides (K_6_ and D_6_) were also measured. Statistically significant differences between LAM-K_6_, LAM-D_6_, PUL-K_6_, PUL-D_6_, and the corresponding native and precursor polysaccharides and peptides were found for (*) *p* < 0.01.

Then, the net electrical charge of the aqueous solutions of the native and polysaccharide derivatives was investigated by measuring their ζ-potentials (Figure 6). The aqueous solutions of LAM, LAM-N_3_, PUL, and PUL-N_3_ exhibited a negligible negative charge of −5.6 ± 0.6 mV, −5.0 ± 0.6 mV, −3.9 ± 0.9 mV, and −1.8 ± 0.3 mV, respectively. However, the conjugation of the negatively charged peptide-bearing alkyne moieties (peptide-D_6_, −10.6 ± 1.7 mV), to either PUL-N_3_ or LAM-N_3_, induced a significant decrease in the net electrical charges of the biopolymers toward more negative values, respectively −24.2 ± 1.8 mV (PUL-D_6_) and −24.5 ± 0.1 mV (LAM-D_6_).On the other hand, the conjugation of either LAM-N_3_ or PUL-N_3_ to the positively charged peptide having alkyne groups (peptide-K_6_, +11.7 ± 1.9 mV) led to an increase in the charge density of the biopolymers toward more positive values, respectively +24.2 ± 2.4 mV (LAM-K_6_) and +19.4 ± 0.9 mV (PUL-K_6_). These results confirm the cationic (LAM-K_6_, PUL-K_6_) and anionic (LAM-D_6_, PUL-D_6_) nature of the polysaccharide-peptide conjugates. 

We hypothesize that such chemical modifications could enable the LbL build-up of new LAM-D_6_/LAM-K_6_ and PUL-D_6_/PUL-K_6_ multilayered coatings at physiological conditions by exploring the electrostatic interactions between the assembling biopolymeric materials exhibiting opposite charges. CHT is the only positively charged naturally occurring polysaccharide and is insoluble at pH > 6.5 [48]. On the other hand, LAM-K_6_ or PUL-K_6_ are both positively charged and water-soluble at physiological pH. Moreover, in opposition to what happens with cationic and anionic natural-origin polysaccharides, the charge density of these polysaccharide-peptide conjugates can be finely tuned by playing with the DS of azide moieties on the polysaccharide backbone or by modifying the peptide sequence.

### 2.2. Build-up of Multilayered Thin Films

**Figure 7:**
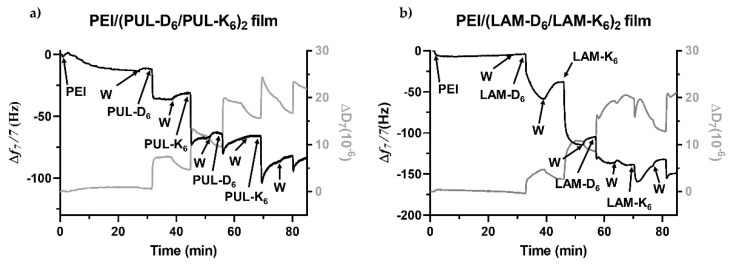
QCM-D monitoring of the LbL deposition of the two bilayered multilayered films onto PEI-functionalized Au-coated quartz crystal sensors and intermediate rinsing steps (W): (**a**) (PUL-D_6_/PUL-K_6_)_2_, and (**b**) (LAM-D_6_/LAM-K_6_)_2_. The graphics represent the changes in frequency (Δ*f_n_*/*n*) and dissipation (Δ*D_n_*) obtained at the seventh overtone (*n* = 7; 35 MHz) as a function of time.

The successful interaction and build-up of multilayered thin coatings encompassing either LAM or PUL coupled to oppositely charged peptides were assessed in situ by the QCM-D technique. This technique is more than a mass sensing device since it allows us to not only detect minute changes in the hydrodynamic mass (ng.cm^−2^) due to changes in the resonance frequency but also measures the viscoelastic properties of the adsorbed layers through the energy dissipated in the mechanical oscillation of the sensors [49]. The growth of multilayered thin films is typically shown by a step-by-step decrease in the normalized frequency shift (Δ*f_n_*/*n*) and an increase in the dissipation factor (Δ*D_n_*) over time after the adsorption of each layered material. As shown in Figure 7a, the normalized frequency shift at the 7th overtone (Δ*f*_7_/*7*) experienced a sequential decrease after the adsorption of the negatively (PUL-D_6_) and positively charged (PUL-K_6_) biopolymer derivatives onto the PEI-functionalized Au-coated quartz crystal substrate. Such behavior reveals not only the deposition of each layered biopolymeric material and, thus, an increase in the adsorbed mass at each deposition step but also the successful interaction and LbL growth of (PUL-D_6_/PUL-K_6_)_2_ multilayered thin coatings. Moreover, the sequential increase in the Δ*D_7_* illustrates the viscoelastic nature of the adsorbed polymeric layers, which is a common feature of soft and hydrated polymeric materials [50]. Similar behavior was denoted for the assembly of oppositely charged LAM-D_6_ and LAM-K_6_ into (LAM-D_6_/LAM-K_6_)_2_ multilayered coatings onto PEI-functionalized surfaces (Figure 7b); however, the decrease in the Δ*f*_7_/*7* right after the adsorption of either the second PUL-D_6_ and PUL-K_6_ (Figure 7a) or LAM-K_6_ layers (Figure 7b) experienced an overshoot, i.e., an increase in the Δ*f*_7_/*7* with an increase in the adsorbed mass. We hypothesize that such behavior might be due to conformational changes in the multilayered film induced by the adsorption of these layered materials on the surface in a tightly packed manner, leading to the collapse of the film due to the removal of coupled water molecules from the adsorbed layers. In addition, one can denote a different behavior in the multilayered growth after the rinsing steps. The rinsing steps in-between the deposition of PUL-D_6_ and PUL-K_6_ produced negligible changes in both the Δ*f*_7_/*7* and Δ*D_7_*, thus revealing the strong association between the assembled polymeric material and the formation of a stable (PUL-D_6_/PUL-K_6_)_2_ multilayered thin film. However, a decrease, in moduli, in the Δ*f*_7_/*7* toward more positive values was noted during the rinsing steps after the deposition of LAM-D_6_ and LAM-K_6_, thus suggesting a decrease in the adsorbed mass and a weaker adsorption process. We hypothesize that such behavior could be assigned to the low-molecular-weight of the assembled LAM-peptide materials when compared to the PUL-peptide counterparts, to the rearrangement of the previously adsorbed layered materials, or to their possible fast diffusion out of the film, as previously reported [51]. Moreover, the key role of electrostatic interactions in the step-by-step growth of both multilayered thin films was unveiled while attempting the assembly of PUL/PUL and LAM/LAM multilayered films, in which there is a clear lack of adsorption of each layered material onto the PEI-functionalized Au-coated quartz substrate after the adsorption of the first layer (Figure S3). The assessment of the morphological properties and in vitro biological performance was performed with the PUL-based multilayered coating since it revealed to be the most stable LbL film.

### 2.3. Scanning Electron Microscopy (SEM)

The assembly of the PUL-peptide films onto the PEI-functionalized Au-coated glass substrates was assessed by SEM-EDS. To perform the SEM-EDS analysis, freshly prepared PUL-peptide films were constructed at 5 mg.mL^−1^ on top of the Au substrate following a procedure reminiscent of the QCM-D experiment, where it was possible to confirm the presence of the multilayer film on the Au substrate (Figure S4). These results corroborate the QCM-D experiment, proving once again that despite the several washing steps, PUL-D_6_ and PUL-K_6_ interact with each other through electrostatic interactions and can form a confluent film when constructed on top of the PEI-functionalized Au-coated glass substrate. Although the SEM-EDS images did not show a uniform and homogeneous biopolymeric film, this was an expected outcome due to the low number of bilayers.

### 2.4. In Vitro Live/Dead Assays

**Figure 8:**
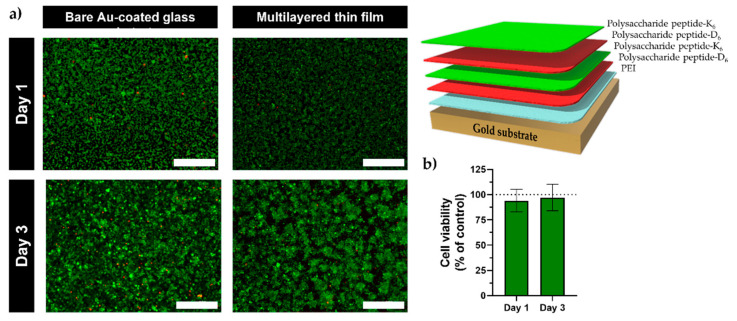
(**a**) Live/dead fluorescence microscopy micrographs of L929 mouse fibroblast cells seeded for 1 and 3 days on the bare (control) and (PUL-D_6_/PUL-K_6_)_2_-coated PEI-functionalized Au substrates (schematic illustration of the multilayered film growth is shown on top-right). The cells were stained in green (live cells) for calcein-AM and in red (dead cells) for propidium iodide (PI). Scale bars = 400 µm. (**b**) Quantification of the L929 mouse fibroblast cells’ viability after 1 and 3 days of culture on top of the multilayered thin films (expressed as a percentage of the control).

The cytocompatibility of the PUL-peptide multilayered films was studied by fluorescence microscopy via a live/dead assay (Figure 8a) and further quantified using ImageJ (Figure 8b). Figure 8a clearly shows that, similarly to the bare Au substrate, the cells seeded on top of the PUL-peptide multilayered thin films remained viable after 72 h (97 ± 13%), indicating no cytotoxicity toward this specific cell line. This assay clearly shows the biocompatibility of the PUL thin films, which is a crucial feature aimed at biomedical applications.

## 3. Materials and Methods

LAM (weight-average molecular weight 5–6 kDa), from *Eisenia bicyclis,* and PUL (weight-average molecular weight 100–200 kDa), from yeasts and fungi, were purchased from Carbosynth (Berkshire, UK). Dry dimethyl sulfoxide (DMSO, p.a. ≤0.02% water) was acquired from Sigma-Aldrich (Taufkirchen, Germany). Sodium azide and 1,1’-carbonyldiimidazole (CDI) were purchased from TCI Chemicals (Zwijndrecht, Belgium). 3-bromopropanol was purchased from Biosynth (Berkshire, UK) and (Tris[(1-benzyl-1H-1,2,3-triazol-4-yl)methyl]amine) (TBTA) was bought from Fisher Scientific (Waltham, MA, USA). The two different peptides were bought from PepMic Co. Ltd. (Suzhou, China) and synthesized in an automated peptide synthesizer, through the standard solid phase peptide synthesis (SPPS), using Fmoc/OtBu strategy. The alkyne functional group required for the conjugation was incorporated during SPPS, using propylic acid reagent. The peptides were then cleaved from the resin already unprotected and purified by high-performance liquid chromatography and identified by mass spectrometry.

The peptides have an alkyne terminal, covalently bonded to a glycine residue, plus six charged amino acids units, either lysine (K) for positive charge (peptide K_6_, with a sequence alkyne-GKKKKKK, MW = 1008.14 g/mol) or aspartic acid (D) for negative charge (peptide D_6_, with a sequence alkyne-GDDDDDD, MW = 929.52 g/mol). A modified PBS composition was used for the QCM-D studies and zeta-potential values acquisition. Briefly, to 100 mL of distilled water, 19 mL of NaH_2_PO_4_.H_2_O (0.2 M), and 81 mL of Na_2_HPO_4_.2H_2_O (0.2 M) were added, the pH was adjusted to 7.4, and the final volume was set to 200 mL. Unless otherwise specified, all chemicals were used as received without further purification.

### 3.1. Synthesis of 3-azidopropanol (AP) and 3-azidopropyl Carbonylimidazole (AP-CI)

The 3-azidopropanol (AP) and 3-azidopropyl carbonylimidazole (AP-CI) were synthetized based on a previously reported work with some modifications [38].

#### 3.1.1. Synthesis of 3-azidopropanol (AP)

Briefly, 3-bromopropanol (5.0 g; 36 mmol) and sodium azide (3.83 g; 59 mmol) were dissolved in 70 mL of a mixture of acetone and water (6:1) and stirred at 65 °C overnight. Acetone was then removed under reduced pressure. Next, 50 mL of water was added to the solution and extracted three times with 50 mL of diethyl ether and the organic phase was dried over magnesium sulfate. Lastly, diethyl ether was removed by rotary evaporation and AP was obtained as a colorless oil (2.5 g, η = 69.4%); ^1^H NMR (300.13 MHz, CDCl_3_): δ (ppm) 1.84 (p, 2H, *J* = 6.4 Hz, C-CH_2_-C), 3.46 (t, 2H, *J* = 6.6 Hz, CH_2_-N_3_), 3.76 (t, 2H, *J* = 6.0 Hz, CH_2_-OH).

#### 3.1.2. Synthesis of 3-azidopropyl Carbonylimidazole (AP-CI)

A dry round-bottomed flask was loaded with 18.5 g (114 mmol) of CDI and 200 mL ethyl acetate, giving rise to a turbid suspension. AP (7.1 mL, 76 mmol) was added dropwise under vigorous stirring while the reaction mixture turned into a clear solution. After 2 h at room temperature, the solution was extracted three times with 200 mL of water. Lastly, the organic layer was dried over magnesium sulfate and evaporated by rotary evaporation and AP-CI was obtained as a pale yellow oil (12.1 g, η = 65.3%); ^1^H NMR (300.13 MHz, DMSO-*d*_6_): δ (ppm) 1.94–2.03 (m, 2H, C-CH_2_-C), 3.55 (t, 2H, *J* = 6.7 Hz, CH_2_-N_3_), 4.43 (t, 2H, J = 6.1 Hz, CH_2_-O), 7.06–7.09 (m, 1H, C=CH-N), 7.61–7.65 (m, 1H, N-CH=C), 8.28–8.32 (m, 1H, N-CH=N).

### 3.2. Synthesis of Polysaccharide-azidopropylcarbonate (LAM-N_3_ and PUL-N_3_)

LAM backbone was modified with azide groups (LAM-N_3_), as described elsewhere [38]. The same methodology was adapted to synthesize PUL-N_3_. Briefly, 1.0 g of polysaccharide, LAM (1.3 mmol) or PUL (1.9 mmol), was dissolved in 20 mL anhydrous DMSO in a dry round-bottomed flask. To this mixture, 214 mg (1.1 mmol) of AP-CI was added and the reaction was stirred overnight at 50 °C under N_2_ atmosphere. The reaction solution was transferred to a dialysis bag with a molecular weight cut-off (MWCO) of 3.5 kDa and dialyzed for 5 days against distilled water, while the dialysis solvent was changed twice a day. After the dialysis was complete the product was frozen at −80 °C and freeze-dried to obtain the purified product as a white fluffy powder that was stored in a dry place protected from light. The DS (number of azide moieties per 100 glucose units) was calculated according to previous reports [44,48]). LAM-N_3_ was synthesized with a DS of 17%; ^1^H NMR (300.13 MHz, D_2_O): δ (ppm) 1.98 (br s, 2H, CH_2_-N_3_), 3.15–4.22 (m, LAM backbone), 4.22–4.31 (m, 2H, CH_2_-O), 4.48–4.62 (m, LAM backbone). PUL-N_3_ was synthesized with a DS of 21%; ^1^H NMR (300.13 MHz, D_2_O): δ (ppm) 1.88–2.20 (m, 2H, CH_2_-N_3_), 3.41–4.03 (m, PUL backbone), 4.19–4.31 (m, 2H, CH_2_-O), 4.92–5.57 (m, PUL backbone).

### 3.3. Synthesis of Polysaccharide–Peptide Conjugates

The peptides K_6_ (alkyne-GKKKKKK) and D_6_ (alkyne-GDDDDDD) were coupled to LAM-N_3_ and PUL-N_3_ via CuAAC. Briefly, 50 mg of azide-polysaccharide, LAM-N_3_ (64.3 μmol) or PUL-N_3_ (96.9 μmol), was dissolved in a mixture of H_2_O:DMSO (1:1). After the complete dissolution of the polysaccharide, 0.25 equiv. of the chosen peptide (K_6_ or D_6_) were added to the reaction mixture. A 500 µL aliquot of CuSO_4_.5H_2_O (0.5 equiv.) was mixed with another 500 µL aliquot of sodium ascorbate (0.5 equiv.) and added to the reaction mixture followed by the addition of a catalytic amount of TBTA. The reaction was performed under stirring at 40 °C for 24 h. Then, the solution was transferred to a dialysis bag with MWCO of 3.5 kDa and dialyzed for 5 days against distilled water, while the dialysis solvent was changed twice a day. After the dialysis was complete, the obtained product was frozen at −80 °C and freeze-dried to obtain the purified compound.

#### 3.3.1. LAM-Peptide Conjugation: LAM-D_6_ and LAM-K_6_

Briefly, 50 mg of LAM-N_3_ was dissolved in a mixture containing 2 mL of ultrapure H_2_O and 2 mL of DMSO. Then, 15 mg of peptide-D_6_ (16.1 μmol) or 16 mg of peptide-K_6_ (16.1 μmol) was added to the reaction mixture. A 500 µL aliquot of a CuSO_4_.5H_2_O aqueous solution (16 mg.mL^−1^) was added to an ice-cold solution (500 µL) of sodium ascorbate (12.7 mg.mL^−1^). After the complete dissolution of the peptide, the 1 mL mixture containing CuSO_4_.5H_2_O and sodium ascorbate was added to the solution, proceeding with an addition of a catalytic amount of TBTA, and the reaction was allowed to stir at 40 °C for 24 h. The reaction mixture was dialyzed and the product was frozen at −80 °C and freeze-dried to obtain the purified LAM peptide as a fluffy powder and stored in a dry place protected from light. LAM-D_6_ ^1^H NMR (300.13 MHz, D_2_O): δ (ppm) 1.98 (br s, 2H, CH_2_-N_3_), 2.93 (br s, peptide D_6_), 3.30–3.92 (m, LAM backbone), 4.18–4.29 (m, LAM backbone), 4.52 (br s, LAM backbone), 8.50 (br s, 1H, triazol-H). LAM-K_6_ ^1^H NMR (300.13 MHz, D_2_O): δ (ppm) 1.32–1.98 (m, peptide K_6_), 2.99 (br s, peptide K_6_), 3.30–3.93 (m, LAM backbone), 4.19–4.29 (m, LAM backbone), 4.52 (br s, LAM backbone), 8.50 (br s, 1H, triazol-H).

#### 3.3.2. PUL-Peptide Conjugation: PUL-D_6_ and PUL-K_6_

Briefly, 50 mg of PUL-N_3_ was dissolved in a mixture containing 2 mL of ultrapure H_2_O and 2 mL of DMSO. Then, 23 mg of peptide-D_6_ (24.2 µmol) or 24 mg of peptide-K_6_ (24.2 µmol) were added to the reaction mixture. A 500 µL aliquot of CuSO_4_.5H_2_O aqueous solution (24.2 mg.mL^−1^) was added to an ice-cold solution (500 µL) of sodium ascorbate (19.2 mg.mL^−1^). After the complete dissolution of the peptide, the 1 mL mixture containing CuSO_4_.5H_2_O and sodium ascorbate was added to the solution, then a catalytic amount of TBTA was added to the reaction mixture and the reaction was allowed to stir at 40 °C for 24 h. After the dialysis, the product was freeze-dried to obtain the purified PUL peptide as a fluffy powder and stored in a dry place protected from light. PUL-D_6_^1^H NMR (300.13 MHz, DMSO-d_6_): δ (ppm). 1.87 (br s, PUL backbone), 4.14–5.01 (m, PUL backbone + peptide D_6_), 5.42–5.58 (m, PUL backbone), 8.08–8.61 (m, peptide D_6_ + triazol-H). PUL-K_6_^1^H NMR (300.13 MHz, D_2_O): δ (ppm) 1.46 (br s, peptide K_6_), 1.68–1.98 (m, peptide K_6_), 2.98 (br s, peptide K_6_), 3.47–4.31 (m, PUL backbone), 5.35–5.38 (m, PUL backbone), 8.59 (s, 1H, triazol-H).

### 3.4. Zeta-Potential Measurements

The zeta-potential (ζ-potential) of the polysaccharide-peptide conjugates, as well as of native polysaccharides and peptides, was determined at 25 °C using a Zetasizer Nano-ZS (Malvern Instruments Ltd., Royston, Hertfordshire, UK). Fresh aqueous solutions (PBS, pH = 7.4) of each component were prepared at 0.5 mg.mL^−1^. Three independent measurements were performed and averaged for each material and reported as mean ± standard deviation. All data were statistically evaluated employing GraphPad Prism software, version 8.0.1 (GraphPad Software Inc, New York, NY, USA), and statistical differences were considered significant when *p*-value < 0.05, established by unpaired *t*-tests.

### 3.5. Quartz Crystal Microbalance with Dissipation Monitoring (QCM-D)

The electrostatic-driven LbL build-up of either LAM-D_6_/LAM-K_6_ or PUL-D_6_/PUL-K_6_ multilayered films was monitored in situ by the fully automated QCM-D apparatus (QSense Pro, Biolin Scientific, Gothenburg, Sweden) in a liquid environment. Gold-coated 5 MHz AT-cut quartz sensors (QSX301 Gold, Q-Sense, Gothenburg, Sweden) were used as substrates after being cleaned with a solution consisting of a 1:1:5 (*v*/*v*) mixture of NH_4_OH (25%), H_2_O_2_ (30%), and ultrapure water in an ultrasonic bath at 70 °C for 10 min, rinsed thoroughly with ultrapure water at room temperature and dried under a soft stream of N_2_. The freshly cleaned Au-coated quartz sensors were inserted in the QCM-D chamber, equilibrated in an aqueous solution (0.1 M PBS pH 7.4) until a stable baseline was achieved, and further functionalized with a polyethyleneimine (PEI) base layer by being exposed to a PEI solution at 0.5 mg mL^−1^ in 0.1 M PBS at pH 7.4 for 25 min. Then, the PEI-functionalized substrates were alternately exposed to aqueous solutions of either negatively (LAM-D_6_ or PUL-D_6_) or positively charged conjugates (LAM-K_6_ or PUL-K_6_), respectively, at 1 mg.mL^−1^ in 0.1 M PBS at pH 7.4 for 6 min each until reaching the adsorption of two LAM-D_6_/LAM-K_6_ or PUL-D_6_/PUL-K_6_ bilayers. In between the deposition of each layered material, a washing step (4 min) was employed to remove weakly adsorbed molecules. The Au-coated quartz sensors were excited at several overtones (*n* = 1st, 3rd, 5th, 7th, 9th, 11th, and 13th, corresponding to 5, 15, 25, 35, 45, 55, and 65 MHz, respectively), and shifts in the frequency (Δ*f_n_/n*) and energy dissipation (Δ*D_n_*) were recorded in real-time. The results shown in this work correspond to the Δ*f* and Δ*D* associated with the 7^th^ overtone (35 MHz), Δ*f_7_/7* and Δ*D_7_*, respectively, after being normalized to the fundamental resonant frequency (5 MHz) of the quartz crystal substrate. However, the results were in good agreement with the other overtones. The adsorption process was performed at 25 °C and a constant flow rate of 50 µL.min^−1^.

### 3.6. Preparation of Multilayered Thin Films onto Gold-Coated Glass Substrates

PUL films were manually assembled in Au-coated glass substrates (1 cm^2^) in a procedure reminiscent of the one described above for the QCM-D experiments for being used in the morphological and in vitro cell studies. Briefly, the Au-coated glass substrates were functionalized with a PEI layer by immersion in a 0.5 mg mL^−1^ solution in 0.1 M PBS at pH 7.4 for 25 min. Then, the PEI-functionalized substrates were alternatively immersed in aqueous solutions of PUL-D_6_ and then PUL-K_6_, respectively, in 0.1 M PBS at pH 7.4 for 6 min each. The adsorption process was repeated twice to enable the build-up of two PUL-D_6_/PUL-K_6_ bilayers onto the PEI-functionalized substrates. After the adsorption of each layered material, a washing step (4 min) was employed to remove unbound molecules. In order to obtain a more homogeneous deposition on top of the substrates, higher biopolymer concentrations were used for the SEM-EDS analysis (5 mg.mL^−1^) and in vitro assays (2 mg.mL^−1^).

### 3.7. Scanning Electron Microscopy (SEM)

Scanning electron microscopy (SEM) imaging of the dried PUL multilayered thin films adsorbed onto PEI-functionalized gold-coated glass substrates was performed in a field emission gun scanning electron microscope (Hitachi SU-3800, Tokyo, Japan) equipped with a Bruker Quantax Compact 30 EDS detector operating in the secondary electrons mode at an accelerating voltage of 15 kV. The gold-coated glass substrates containing the adsorbed layers were fixed onto a metal stub by double-sided carbon conductive tape for enabling electrical contact and the analysis was attempted in high vacuum.

### 3.8. In Vitro Assays: Cell Seeding and Live-Dead Staining

L929 mouse fibroblasts (ATCC ^®^ CRL-6364™) were seeded in tissue culture flasks T-175 (Sarstedt) containing low glucose DMEM supplemented with 10% FBS and 1% antibiotic/antimycotic (penicillin/streptomycin) in a humidified incubator at 37 °C and 5% CO_2_. Upon reaching confluence, cells were trypsinized and seeded (1.5 × 10^4^ cells/film) on top of the PEI-functionalized gold-coated glass substrates containing the PUL multilayered films on 12-well plates for 24 and 72 h. As control experiment, the same cell density was seeded on bare gold-coated glass substrates. Cell viability was evaluated by incubating the assembled films with Calcein-AM/propidium iodide (PI) (live-dead Kit, ThermoFischer Scientific, Waltham, MA, USA) for 20 min, according to the manufacturer’s protocol. Widefield fluorescence analysis was performed in a Zeiss Imager M2 widefield microscope equipped with a 3Mpix camera and a 10x objective (Carl Zeiss Microscopy, Oberkochen, Germany). Acquired data were processed in Zeiss ZEN v2.3 blue edition software. In addition, the cell viability was quantified by fluorescence intensity using the ImageJ software, version Fiji 1.52n (ImageJ Software, Bethesda, MD, USA), and the data were statistically evaluated employing GraphPad Prism software, reported as mean ± standard deviation (*n* = 3).

## 4. Conclusions

Herein we report a novel approach to enable the assembly of neutral marine biopolymers into electrostatic-driven multilayered thin films by covalently grafting positive and negatively charged low-molecular-weight molecules bearing alkyne groups to the azide-functionalized biopolymers, followed by their assembly into innovative multilayered coatings. The employed strategy resorted to the highly selective CuAAC chemistry to conjugate small peptide sequences, exhibiting charged amino acids, to neutral polysaccharides, thus turning them into charged biopolymers. The successful coupling of the peptides to the polysaccharide backbone was revealed by ^1^H NMR and ATR-FTIR spectroscopy. Moreover, zeta-potential measurements confirmed the functionalization of the marine polysaccharides with the peptide sequences, as shown by the change in the net electrical charge of the polysaccharide derivatives when compared to the native polysaccharides. The assessment of the multilayered film growth was performed in situ by the QCM-D technique, revealing that both the PUL- and LAM-derived multilayered thin coatings can be assembled. However, a stronger association between the LbL ingredients and stable build-up was found for the assembly of the PUL-peptide conjugates when compared to the LAM-peptide counterparts, most likely due to the higher molecular weight of PUL. The versatility imparted by such an approach would enable the functionalization of virtually any uncharged biopolymer with any kind of charged small (bio)molecules to turn them into charged biopolymeric materials and enable their assembly into a multitude of electrostatic-driven multilayered structures and devices denoting fine-tuned properties and multifunctionality at the nanoscale. For instance, the biopolymer functionalization with cell-adhesive domains (e.g., arginine-glycine-aspartic acid (RGD)) or MMP-sensitive peptide sequences would impart the resulting multilayered assemblies with bioinstructive or enzyme-sensitive properties, thus opening new avenues in controlled drug/therapeutics delivery, tissue engineering, and regenerative medicine strategies.

## Data Availability

Not applicable.

## Supplementary Materials

The following supporting information can be downloaded at: https://www.mdpi.com/article/10.3390/md21020092/s1, Figures S1 and S2: ^1^H NMR spectra; Figure S3: QCM-D build-up of multilayered assemblies; Figure S4: SEM-EDS images.
