# Supplementary material for: Unveiling the Assembly of Neutral Marine Polysaccharides into Electrostatic-Driven Layer-by-Layer Bioassemblies by Chemical Functionalization
Source: Mar Drugs. 2023 Jan 27;21(2):92. doi: 10.3390/md21020092 (PMC9964173; doi:10.3390/md21020092)
Supplement: Supplementary file 1 [file marinedrugs-21-00092-s001.zip › marinedrugs-2101364-supplementary.pdf]

Unveiling the Assembly of Neutral Marine Polysaccharides into Electrostatic-Driven Layer-by-Layer bioassemblies by Chemical Functionalization

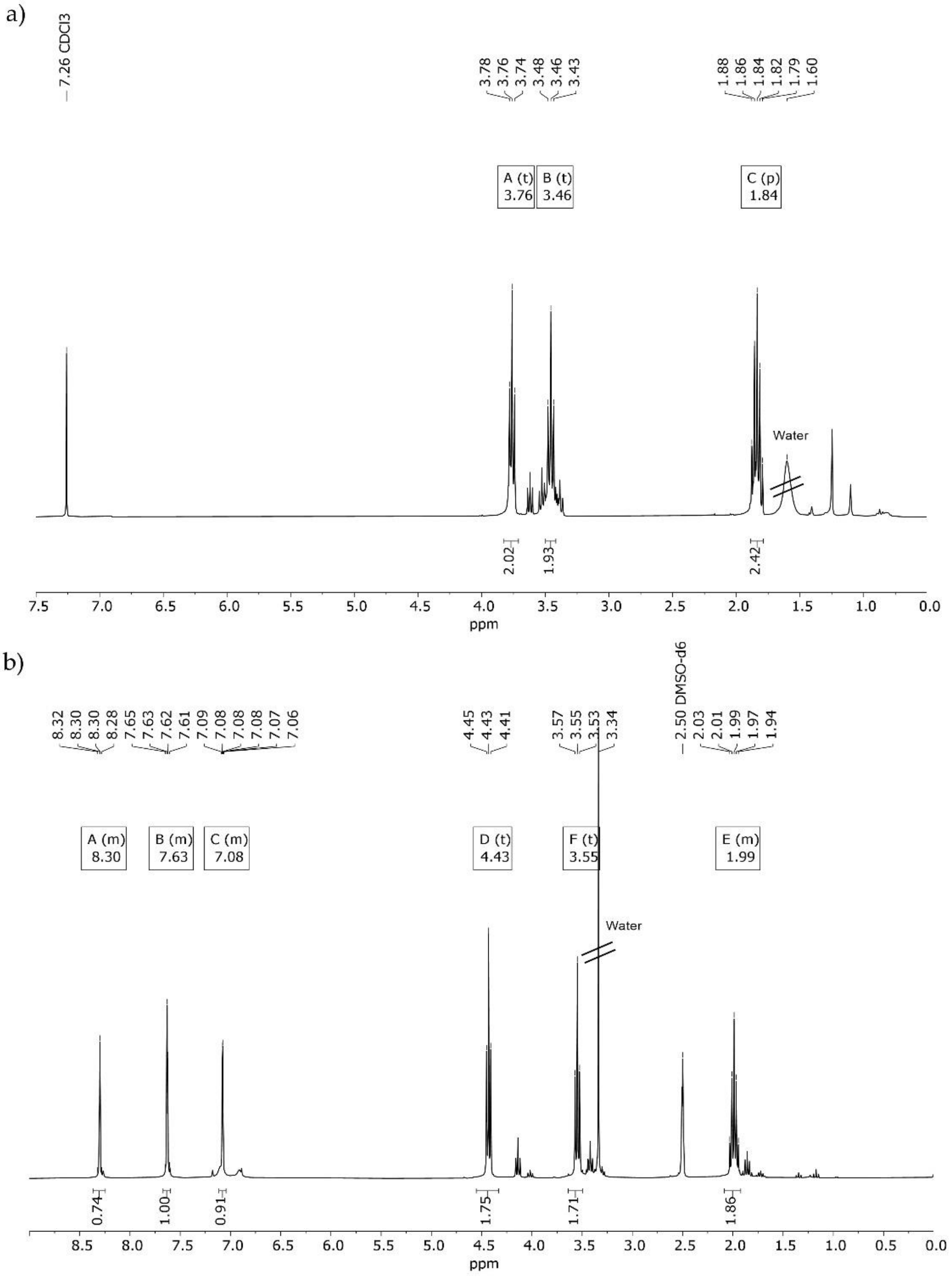

Figure S1: ^{1}H NMR spectra of (a) propanol azide in $CDCl_3$ and (b) CDI-azide in DMSO-d_6.

a)

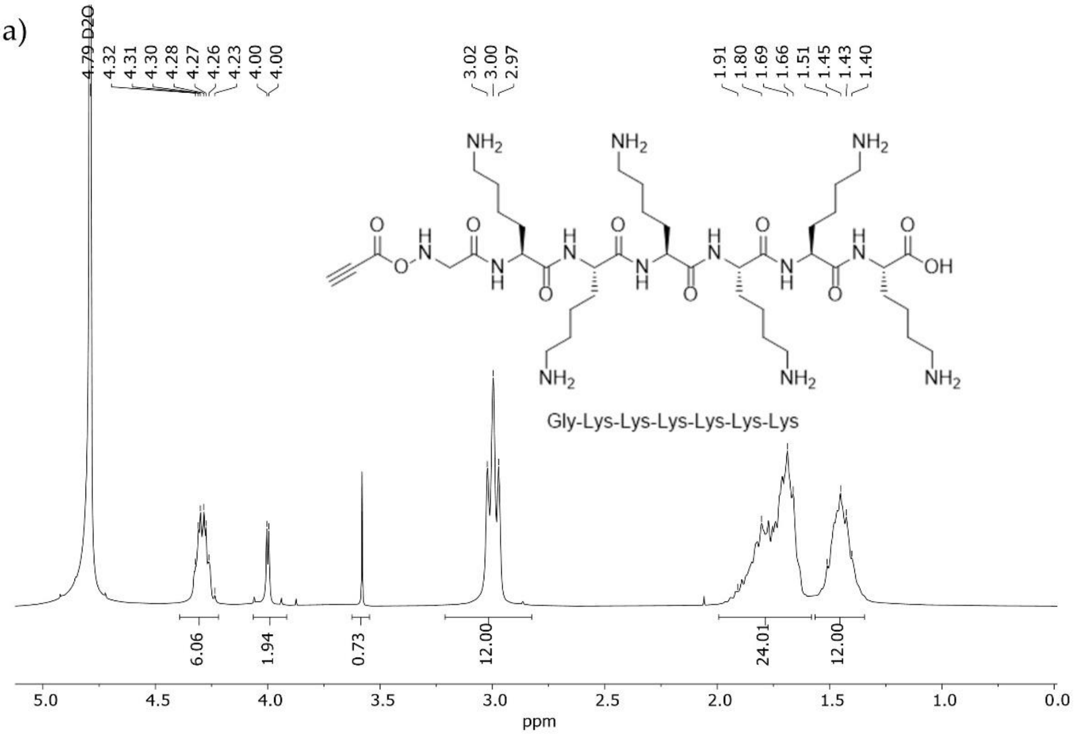

b)

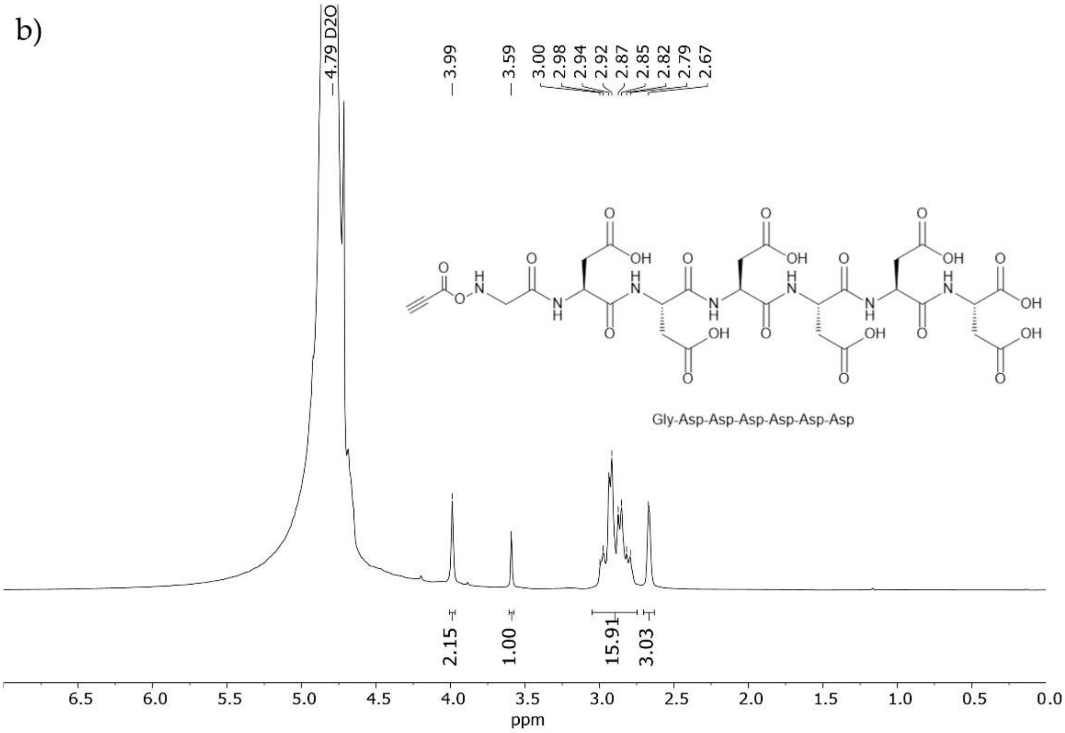

Figure S2: ^{1}H NMR spectra of the peptides (a) K_6 and (b) D_6, in D_2O.

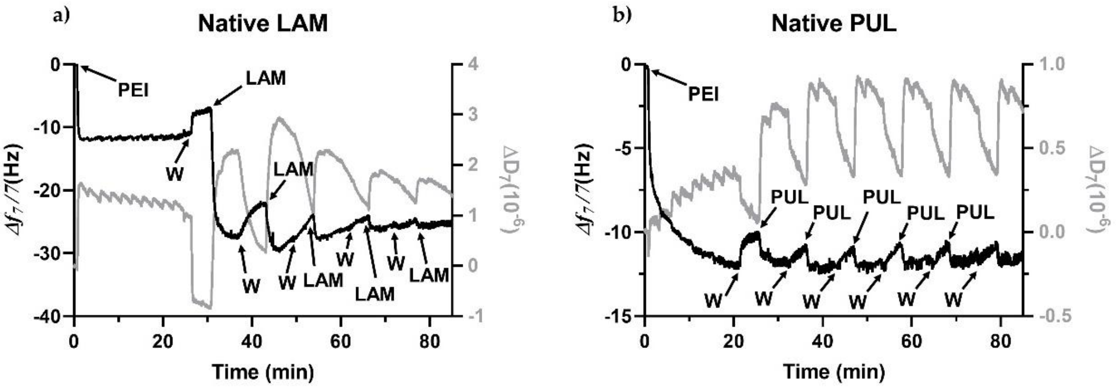

Figure S3: QCM-D build-up assessment of the LbL build-up of (a) $(LAM/LAM)_2$ and (b) $(PUL/PUL)_2$ multilayered films onto PEI-functionalized quartz crystal sensors and intermediate rinsing steps (W). The graphics represent the changes in the frequency ($\Delta f_n/n$) and dissipation (ΔD_n), obtained at the seventh overtone (n = 7; 35 MHz), as a function of time.

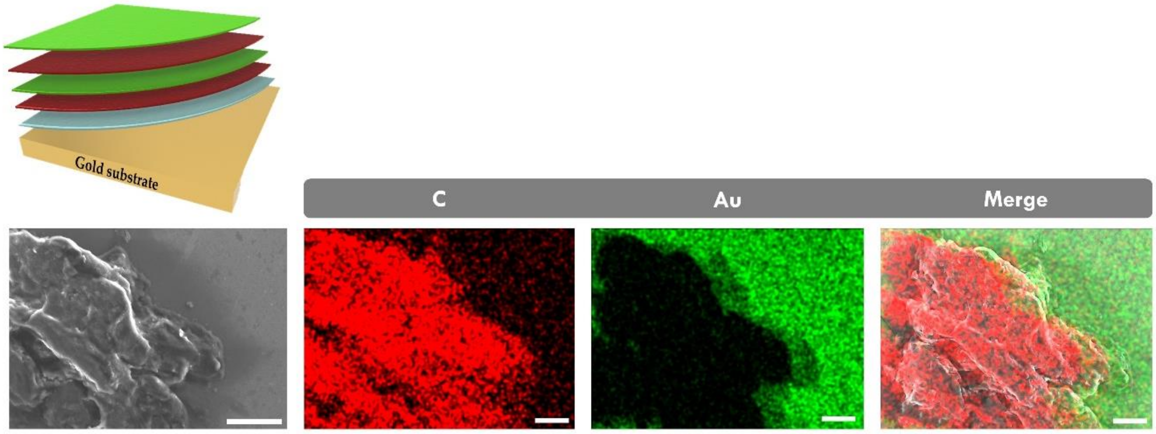

Figure S4: SEM-EDS images of the PUL-peptide films (5 mg.mL^{-1}) constructed onto PEI-functionalized Au-coated glass substrates highlighting the presence of carbon (C) in red, gold (Au) in green, and the merge image of both C and Au. Scale bars = 25 μm.
